# Comparison of Metal-Based Nanoparticles and Nanowires: Solubility, Reactivity, Bioavailability and Cellular Toxicity

**DOI:** 10.3390/nano12010147

**Published:** 2021-12-31

**Authors:** Johanna Wall, Didem Ag Seleci, Feranika Schworm, Ronja Neuberger, Martin Link, Matthias Hufnagel, Paul Schumacher, Florian Schulz, Uwe Heinrich, Wendel Wohlleben, Andrea Hartwig

**Affiliations:** 1 Department of Food Chemistry and Toxicology, Faculty of Chemistry and Biosciences, Institute of Applied Biosciences, Karlsruhe Institute of Technology (KIT), 76131 Karlsruhe, Germany; Johanna.Wall@kit.edu (J.W.); fschworm@gmail.com (F.S.); Ronja.Neuberger@kit.edu (R.N.); Martin.Link@kit.edu (M.L.); Matthias.Hufnagel@gmail.com (M.H.); Paul.Schumacher@kit.edu (P.S.); 2 BASF SE, 67063 Ludwigshafen, Germany; Didem.Ag-Seleci@basf.com (D.A.S.); wendel.wohlleben@basf.com (W.W.); 3 Fraunhofer ITEM, 30625 Hannover, Germany; Florian.Schulz@item.fraunhofer.de; 4 ToxConsultant, 30625 Hannover, Germany; uwe.heinrich1512@gmail.com

**Keywords:** metal-based nanoparticles and nanowires, solubility, intracellular bioavailability, oxidative reactivity

## Abstract

While the toxicity of metal-based nanoparticles (NP) has been investigated in an increasing number of studies, little is known about metal-based fibrous materials, so-called nanowires (NWs). Within the present study, the physico-chemical properties of particulate and fibrous nanomaterials based on Cu, CuO, Ni, and Ag as well as TiO_2_ and CeO_2_ NP were characterized and compared with respect to abiotic metal ion release in different physiologically relevant media as well as acellular reactivity. While none of the materials was soluble at neutral pH in artificial alveolar fluid (AAF), Cu, CuO, and Ni-based materials displayed distinct dissolution under the acidic conditions found in artificial lysosomal fluids (ALF and PSF). Subsequently, four different cell lines were applied to compare cytotoxicity as well as intracellular metal ion release in the cytoplasm and nucleus. Both cytotoxicity and bioavailability reflected the acellular dissolution rates in physiological lysosomal media (pH 4.5); only Ag-based materials showed no or very low acellular solubility, but pronounced intracellular bioavailability and cytotoxicity, leading to particularly high concentrations in the nucleus. In conclusion, in spite of some quantitative differences, the intracellular bioavailability as well as toxicity is mostly driven by the respective metal and is less modulated by the shape of the respective NP or NW.

## 1. Introduction

Engineered metal-based nanomaterials are used in many consumer products, such as textiles, electronics, or medicinal products [1,2,3,4]. Besides metal-based nanoparticles, fibrous materials (nanowires) are also gaining increasing attention, e.g., for transparent conductive layers on displays [5,6]. Thus, a wide range of nanomaterials is available, differing in physicochemical properties such as size, shape, and surface chemistry.

Inhalation is the most crucial route of exposure and uptake of nanomaterials, especially at the workplace [7]. Depending on the size and shape, nanomaterials can reach different areas in the lung, including the alveolar region [8]. However, a general toxicological evaluation seems difficult, due to the diversity of these nanomaterials, and considering their broad range of physicochemical properties. Therefore, a grouping approach based on several physicochemical properties appears to be promising to elucidate the toxicological potential of nanomaterials [9,10,11].

For metal-based nanomaterials, the generation of ROS and thus oxidative stress has been identified as an important mechanism leading to genotoxicity and cytotoxicity [12]. Hereby, two scenarios are conceivable. First, the intracellular release of metal ions appears to be of major importance [13,14,15]. The so-called ‘Trojan horse type’ mechanism describes an endocytotic uptake, followed by lysosomal dissolution of the metal-based materials, resulting in high metal ion concentrations within the cells. This observation has already been made for Cu-, Ag-, and Ni-based nanoparticles (NP) [16,17,18,19]. Second, the materials themselves are able to catalyze the formation of ROS, due to their specific surface reactivity. Recent studies already demonstrated a high oxidative reactivity of different metal-based nanoparticles [20].

While there are increasing numbers of studies conducted with particulate nanomaterials, little is known about the reactivity of metal-based nanowires (NW). Depending on the cell type, previous studies indicate that metal-based fibrous nanomaterials are taken up into cells [21,22,23,24]. Furthermore, for Ag NW an uptake by endosomal and lysosomal vesicles has already been postulated [25]. However, the questions whether or not their fibrous structure contributes to toxicity and whether or not metal ions are intracellularly released as well remain. Therefore, within the present study, we comprehensively characterized particulate and fibrous metal-based nanomaterials with respect to their physicochemical properties. Besides nanomaterials known to potentially release toxic metal ions (Cu, CuO, Ni, and Ag), rather non-reactive materials (CeO_2_ and TiO_2_) with particulate and fibrous morphologies were also included. We compared them with respect to abiotic metal ion release in different physiologically relevant media and to acellular reactivity. Subsequently, four different cell lines were applied to compare cytotoxicity as well as intracellular metal ion release in the cytoplasm and nucleus. Since inhalation is likely to be the most critical route of exposure, two human lung epithelial cell lines were chosen, representing different regions of the respiratory tract, namely A549 cells (alveolar region) and BEAS-2B (normal human bronchial epithelium obtained from non-cancerous individuals). Furthermore, differentiated THP-1 cells (human peripheral blood monocytes) were chosen as a model for human macrophages. Finally, since inhalation studies are mostly performed in rats, RLE-6TN, an alveolar epithelial cell line derived from the rat lung was included as well, to compare the toxicity and bioavailability derived for human cells to rat cells.

## 2. Materials and Methods

### 2.1. Materials

All materials were purchased (purity > 99%) from Sigma Aldrich (Darmstadt, Germany) or Carl Roth GmbH (Karlsruhe, Germany). The nanomaterials included are listed in Table 1.

### 2.2. Physicochemical Characterization

In a first step, the nanomaterials were dispersed freshly according to the NANOGENOTOX protocol to a concentration of 2.56 mg/mL in 0.05% BSA. Sonication was performed using a Branson Analog Sonifier 450 (Brookfield, CT, USA) for 13:25 min at 10% amplitude (7179 J). After sonication, the stock solution was diluted in supplemented RPMI-1640 to achieve the respective incubation concentrations. To investigate the impact of a freeze-thawing protocol [27] on the particle properties of the NP, samples were treated according to Keller and colleagues prior to DLS analyzes. Briefly, immediately after sonication, samples were frozen in liquid nitrogen at −196 °C. For DLS analyzes, samples were sonicated at 60 °C for at least 1 min or until completely thawed. Subsequently, NP were diluted to 100 µg/mL in supplemented RPMI-1640, incubated at 37 °C for 1 h, and analyzed [27].

Measurements of hydrodynamic diameter, zeta potential, and polydispersity index (PDI) of the NM dispersions were performed at a concentration of 100 µg/mL in RPMI supplemented with 10% FBS using a Zetasizer Nano ZS (Malvern Instruments Ltd., Worcestershire, UK).

Electron microscopy to obtain primary particle diameters as well as fiber diameters and lengths was performed in cooperation with the Laboratory for Electron Microscopy at KIT. Here, either a transmission electron microscope (CM200 FEG/ST, Philips, Amsterdam, The Netherlands) for NP or a scanning electron microscope (1530 Gemini with Schottky field emitter, LEO, Zeiss, Oberkochen, Germany) for nanowire was used. Subsequently, at least 300 particles or fibers were analyzed regarding their diameter and length using the ImageJ software (Rasband, National Institutes of Health, Bethesda, MD, USA).

The effective densities of the NP in supplemented RPMI-1640 were obtained as previously described by DeLoid and colleagues [28]. Briefly, 1 mL of the respective sample were transferred into VCM tubes (87007, TPP Techno Plastic Products AG, Trasadingen, Switzerland) and centrifuged for 1 h at 3000× *g*. Subsequently, the volume of the pelleted NP was determined using a VCM “easy read” measuring device (87010, TPP Techno Plastics Products AG, Trasadingen, Switzerland). Effective densities were finally calculated according to De Loid and colleagues [28].

Data on deposited NP was obtained in silico using the Distorted Grid (DG) model as published previously by DeLoid and colleagues [29,30]. The model was performed according to Keller and colleagues using an auxiliary MATLAB macro to integrate data input via Microsoft Excel and allowing batch processing of multiple materials. The applied parameters for modeling particle deposition are listed in Table S1, the respective effective densities are stated in Table 2. The purity of the nanomaterials was determined in cooperation with the Institute of Applied Geosciences at KIT using ICP-MS (X-Series2 with collision cell technology, Thermo Fisher, Langenselbold, Germany).

### 2.3. Cell Culture

A549 cells (human adenocarcinoma cells) were kindly provided by Dr. Roel Schins (Leibniz Research Institute for Environmental Medicine, Düsseldorf, Germany). A549 cells were cultured in RPMI-1640 with 10% FBS, 100 U/mL penicillin, and 100 µg/mL streptomycin.

THP-1 cells (human peripheral blood monocytes, ATCC TIN-202) were kindly provided by Dr. Richard Gminski (Albert-Ludwig-University Freiburg, Department of Environmental Health Sciences and Hygiene, Freiburg, Germany) and cultured in supplemented RPMI-1640 composed as described above. Prior to experiments, THP-1 cells were seeded in cell culture dishes and differentiated with 30 ng/mL phorbol 12-myristate 13-acetate (PMA, diluted in DMSO) for 4 days. After 4 days, the differentiated cells (dTHP-1 cells) were cultured for further 3–5 days in fresh cell culture media [31].

RLE-6TN cells (alveolar epithelial cells derived from the rat lung, ATCC CRL-2300) were cultured in Hams-F12 media supplemented with 10% FBS, 100 U/mL penicillin, 100 µg/mL streptomycin, 10 µg/mL bovine pituitary extract, 5 µg/mL bovine insulin, 2.5 ng/mL epidermal growth factor, 2.5 ng/mL insulin like growth factor I and 1.25 µg/mL transferrin. Cells were subcultured twice a week.

Beas-2B cells (human lung bronchial epithelial cells, ATCC CRL-9609) were kindly provided by Dr. Carsten Weiss (Karlsruhe Institute of Technology, Karlsruhe, Germany). They were grown in precoated cell culture dishes (10 µg/mL fibronectin, 30 µg/mL collagen and 10 µg/mL bovine serum albumin in PBS) and cultured in keratinocyte growth media.

All cells were cultured under a humidified atmosphere with 5% CO_2_ in air (HeraSafe, Thermo Scientific, Langenselbold, Germany). Except of the A549 cells, accutase was used instead of trypsin to detach adherent cells. For in vitro experiments, the cell number for each cell line was adjusted to achieve a confluent monolayer at the time of incubation, with the exception of dTHP-1 cells which were not able to form a confluent monolayer and therefore were seeded at the same cell number as the A549 cells.

### 2.4. Dissolution

#### 2.4.1. Static Dissolution

To investigate the solubility of the nanomaterials, the dissolution rate of the materials in different physiologically relevant media as well as in the experimental setup was determined as previously described [15]. Briefly, the nanomaterial stock solution was diluted to a concentration of 100 µg/mL either in artificial alveolar fluid (AAF; pH 7.4 (composed of magnesium chloride (0.0952 g/L), sodium chloride (6.0193 g/L), potassium chloride (0.2982 g/L), disodium hydrogen phosphate (0.1420 g/L), sodium sulfate (0.0710 g/L), calcium chloride dihydrate (0.3676 g/L), sodium acetate trihydrate (0.9526 g/L), sodium hydrogen carbonate (2.6043 g/L), trisodium citrate dehydrate (0.0970 g/L), lecithin (0.1000 g/L)), artificial lysosomal fluid (ALF; pH 4.5 (composed of sodium chloride (3.210 g/L), sodium hydroxide (6.000 g/L), citric acid (20.800 g/L), calcium chloride dihydrate (0.1285 g/L), disodium hydrogen phosphate (0.0710), sodium sulfate (0.0390 g/L), magnesium chloride (0.0476 g/L), glycine (0.0590 g/L), sodium citrate dehydrate (0.0770 g/L), sodium tartrate dihydrate (0.0900 g/L), sodium lactate (0.0850 g/L), sodium pyruvate (0.0860 g/L)), or cell culture media and incubated for 24 h or 168 h at 37 °C and 150 rpm in a centrifuge tube. After incubation, the suspension was centrifuged at 3000× *g* for 1 h, followed by repeated centrifugation of the supernatants at 16,000× *g* for 1 h. Subsequently 2 mL of the supernatant were collected and centrifuged again at 16,000× *g* for 1 h. The supernatant was checked to exclude residual particles via dynamic light scattering and subsequently metal analyzes was performed. Subsequently, 1 mL of the supernatant was heated stepwise to 95 °C to dry up. The remnants were further digested with 1:1 HNO_3_ (69%)/H_2_O_2_ (31%) (*v*/*v*) by repeated stepwise heating to 95 °C. The residue was then solubilized in 1 mL HNO_3_ (0.2%) and metal ion content was measured by either GF-AAS (PinAAcle 900 T, Perkin Elmer, Rodgau, Germany) or ICP-MS (iCAP RQ with collision cell technology, Thermo Fisher, Langenselbold, Germany).

#### 2.4.2. Dynamic Dissolution and Transformation

The flow-through setup which implements a “continuous flow system” (ISO 19057:2017) was used to detect nanoparticles and nanowire dissolution [32]. Briefly, NP/NW mass of 1 mg was weighed onto a membrane (cellulose triacetate, Sartorius Stedim Biotech GmbH, Göttingen, Germany: 47 mm diameter, 5 kDa pore size), topped by another membrane, and enclosed in flow-through cells. The flow-through cells were kept upright within a thermostatically controlled water bath to ensure that emerging air bubbles were able to leave the system without accumulating within the cell. The phagolysosomal simulant fluid (PSF) pH 4.5, which is an acidic buffer simulating the phagolysosomal compartment of macrophages [33], was employed at 37 ± 0.5 °C. The programmable sampler drew 10 mL eluates once per day from the total 100 mL collected. The ion concentration in the eluates was determined by ICP optical emission spectrometry (ICP-OES, Agilent 5100, Agilent Technologies, Santa Clara, CA, USA). After the experiment, the cells were flushed with deionized water before opening them to rinse the remaining solids off the membrane. The resulting suspension was then pelleted onto a transmission electron microscopy (TEM) grid held at the bottom of a centrifuge vial within 30 min and dried subsequently. By this procedure, the morphology of the remaining solids could be inspected with a reduction of interference from drying artifacts of PSF salts, which are removed by this preparation. Particle morphology was analyzed by TEM with a Tecnai G2-F20ST or Tecnai Osiris Microscope (FEI Company, Hillsboro, OR, USA) at an acceleration voltage of 200 keV under bright-field conditions. X-ray photoelectron spectroscopy (XPS) was performed using a Phi Versa Probe 5000 spectrometer (Physical Electronics, Feldkirchen, Germany) applying monochromatic Al Kα radiation.

### 2.5. Abiotic Reactivity (FRAS Assay)

The SOP, which described the multi-dose protocol of the Ferric Reduction Ability of Serum (FRAS) assay, published in 2017 by BASF [34], was used for reactivity testing of samples under physiological conditions.

Briefly, samples were incubated with Human Blood Serum (HBS) for 3 h at 37 °C. Before incubation, bath sonication for 1 min was applied to prevent the formation of large agglomerates and access whole surface area. NMs were separated from HBS via ultracentrifugation (AUC-Beckman XL centrifuge, Beckman Coulter, Brea, CA, USA) at 14,000× *g* for 150 min). Subsequently, 100 μL of NM-free HBS supernatant were incubated in the FRAS reagent that contains the Fe^3+^ complex. The total antioxidant depletion as a measure of the oxidative potential of NMs was determined by using UV-vis spectrum of the iron complex solution. Trolox, a water-soluble analog of vitamin E, was used as an antioxidant to calibrate the FRAS results. Different Trolox concentrations (from 0.001 to 0.1 g/L) were tested by FRAS assay to obtain FRAS absorption signals that were linearly fitted. Finally, the oxidative damage induced by NP and NW was calculated in Trolox equivalent units (TEUs).

Additionally, fresh NM samples were prepared to evaluate the ion contribution. After an ultracentrifugation step, the ion concentration in NM-free HBS supernatants was determined by ICP-MS (Nexion 2000b, Perkin Elmer, Waltham, MA, USA). Using water-soluble metal salts (CuSO_4_·5H_2_O and NiCl_2_), ion solutions with equivalent concentrations were prepared and the associated oxidative damage in HBS was measured by FRAS method. For each NM and ion dose, triplicate measurements were performed.

### 2.6. Cytotoxicity, Bioavailability, and Intracellular Distribution

For cytotoxicity testing, the ATP content was quantified with the CellTiter-Glo^®^ Luminescent Cell Viability Assay Kit (Promega GmbH, Bremen, Germany). Briefly, cells were seeded in 96-well plates and incubated with the respective nanomaterials or 500 nM staurosporine (positive control). After 24 h incubation the medium was removed and 100 µL fresh medium was added to the wells. After 30 min of equilibration, 100 µL of CellTiter-Glo^®^ was added and chemiluminescence was measured on the Infinite^®^ 200 Pro microplate reader (Tecan Group Ltd., Männedorf, Switzerland). For analyzing the relative cell count (RCC) and bioavailability, cells were seeded as a confluent monolayer and incubated with three different doses of the nanomaterials (Table S2). After 24 h, incubation was stopped by removing the incubation media from the cells. The cells were washed with PBS, collected, and analyzed via a Casy cell counter obtaining cell number and cell volume. Cell count was used to determine the RCC. To quantify the amount of bioavailable metal ions within the whole cell, cells were lysed in RIPA buffer (0.01 M Tris pH 7.6, 0.15 M NaCl, 0.001 M EDTA, 1% (*v*/*v*) Triton-X 100, 1% DOC, 0.01% SDS, 1 × protease-inhibitor) for 30 min followed by 1 h centrifugation at 14,000× *g* to remove the cell membrane and undissolved material residues [17]. The supernatant was then used for graphite furnace atomic absorption spectrometry (GF-AAS) as described above. Residues of particles were excluded using dynamic light scattering (data not shown). To investigate the intracellular distribution of the metal ions within the cytoplasm and the nucleus, cells were fractionated as described previously [15,17], using the Nuclear Extract Kit (ActiveMotif, Carlsbad, CA, USA).

## 3. Results

### 3.1. Physicochemical Characterization

All materials were characterized as raw material or dispersion in detail. For all particles, hydrodynamic (d_h_) and primary diameter (d_p_, obtained via TEM or SEM) as well as ζ-potential and PDI were determined. Additionally, all particles were investigated with respect to their specific surface area (SSA), effective density, and deposition efficiency for subsequent in vitro studies using the Distorted Grid (DG) model (Table 2). The primary particle size of all materials ranged between 15.5–55.2 nm, with Ag NP (NM 300K) showing the smallest size followed by CuO NP, Ni NP, CeO_2_ NP (NM 212), and TiO_2_ NP (NM 105). Cu NP showed the largest primary diameter of 55.2 nm. The hydrodynamic diameter of all materials was in the same order of magnitude, except for the Ni NP which showed a high d_h_ of 388 nm. Additionally, a rather high PDI for the Ni NP was observed. The latter, in combination with the high d_h_, indicates that the Ni NP dispersion was very poly-disperse and that this particle species tended to agglomerate. The deposited dose ranged between 22% in case of TiO_2_ NP and 90% in case of Ni NP. To facilitate the dispersion preparation for in vitro studies, a comparison between freshly prepared and thawed material dispersions was made. Hereby, d_h_, Z-average and the deposited dose were investigated (Table S3, Figures S1 and S2). Altogether, no major differences were observed, indicating the applicability of a freeze-thaw protocol. For the fibrous materials, primary length and diameter, as well as ζ-potential and SSA were investigated (Table 3). All nanowire showed a mean length above 5 µm and a width below 500 nm, indicating WHO fibre-like properties with an aspect ratio higher than 3:1. To calculate the deposited dose for the NP, the DG model was used [29]. Since this model uses spherical structures to simulate the deposited dose it is not applicable for fibrous materials such as nanowire.

### 3.2. Dissolution and Transformation

The solubility in physiologically relevant fluids was determined using two approaches, applying a static and a dynamic method. While the static approach was applied for artificial alveolar (AAF, pH 7.4) and artificial lysosomal (ALF, pH 4.5) fluid, the dynamic approach was conducted with a phagolysosomal simulant fluid (PSF, pH 4.5). The results are summarized as a proportion of dissolved material after seven days of incubation (Table 4). Most materials were not soluble in AAF. Only Cu NP and Cu NW showed a low and comparable solubility in AAF of 12%. The solubility in artificial lysosomal fluid (ALF) depended strongly on the material examined. High solubilities were observed in the case of Cu NP (64%) and Cu NW (57%), as well as for CuO NP (57%) in the static method. These materials also showed high solubility in the dynamic process, whereby the CuO NP exerted the highest solubility of 97%. The nickel materials exhibited a moderate (Ni NW) to high solubility (Ni NP) in the static process, while Ni NW showed a high solubility of 94% in the dynamic process. In both, static and dynamic approaches, no or only a very low solubility for the Ag NP was detected. The fibrous silver material (Ag NW) was also found to be insoluble by the static approach, while a low solubility of 11% was found applying the dynamic system. For particulate and fibrous TiO_2_ as well as for particulate CeO_2_ no solubility was observed in any of the media tested.

**Figure 1:**
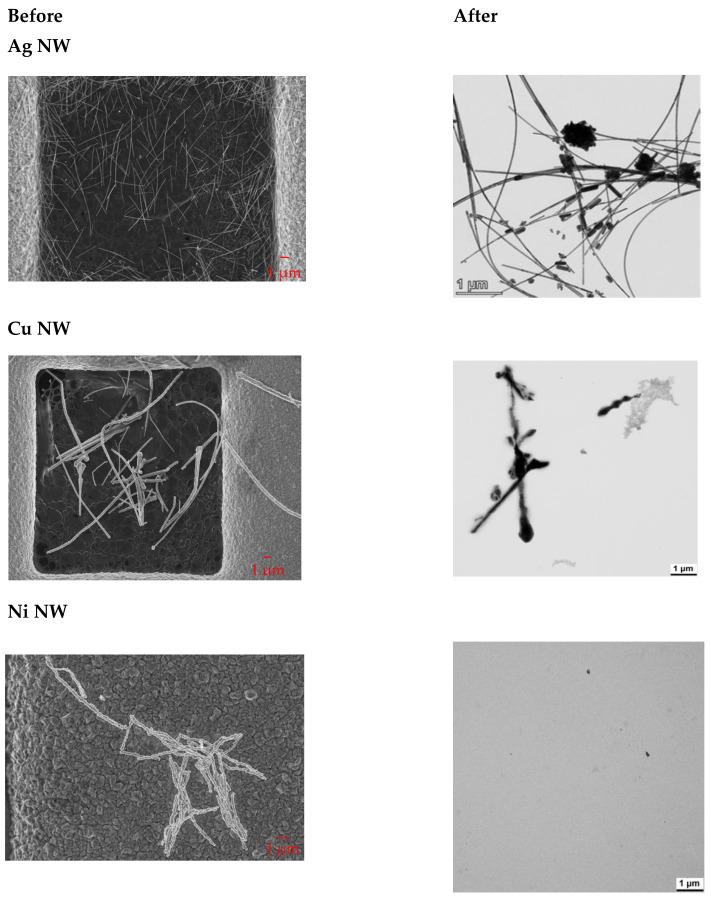

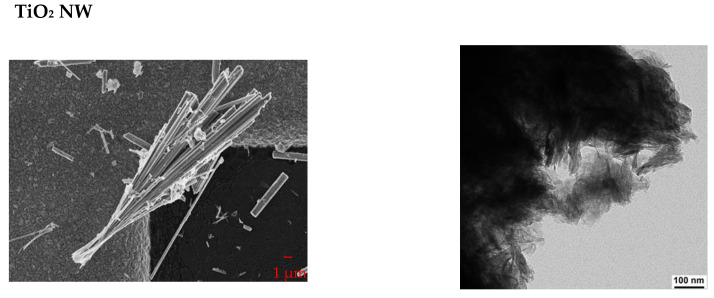
TEM images of NP and NW before and after treatment in the flow-through cells with phagolysosomal simulant fluid (PSF).

Next, the transformation of nanomaterial shape and speciation of NW after dynamic dissolution was investigated (Figure 1). For this purpose, the flow-through cells were flushed with water, opened and the remaining solids were rinsed onto a centrifuge vial with a TEM grid at the bottom. By centrifugation, all solid material > 10 nm was spun onto the TEM grid and the supernatant containing the buffer salts was discarded. Compared to non-treated Ag NW, the occurrence of particulate morphologies was observed, which represents the thermodynamically stable form. A smaller number of Cu NW, decorated by newly formed substructures, was found on TEM grids due to high solubility in PSF. The tendency of increased polymorphism from long fibers towards a higher number of small particle structures coincided with sulfidation (EDXS, data not shown), which may have formed passivating layers. Since Ni NW almost completely dissolved, no NW were detected during TEM measurements. As expected, undissolved TiO_2_ NW stayed aggregated after dissolution.

### 3.3. Abiotic Reactivity

**Figure 2:**
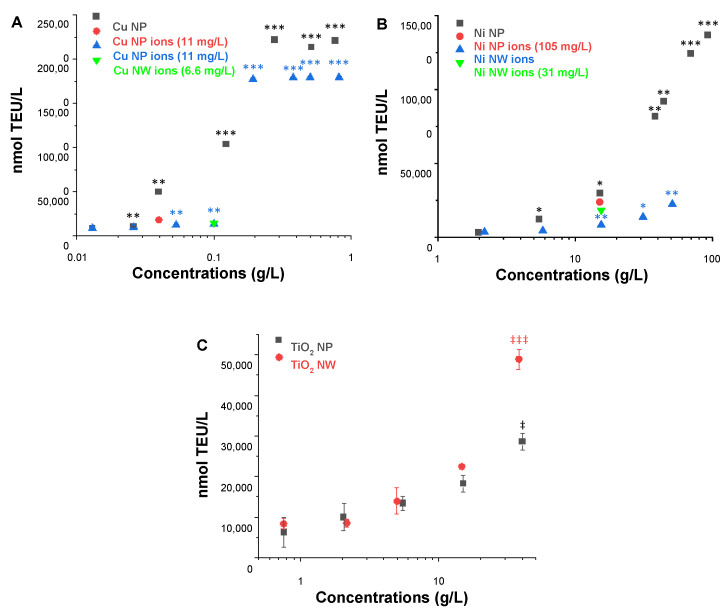
FRAS testing (**A**) Cu NP, Cu NP ions, Cu NW and Cu NW ions (**B**) Ni NP, Ni NP ions, Ni NW and Ni NW ions (**C**) TiO_2_ NP and TiO_2_ NW. Error bars indicate one standard deviation from triplicate testing, and are smaller than the size of the symbol in most cases. Statistics were performed using either ANOVA-Dunnett’s T3 (* *p* ≤ 0.05, ** *p* ≤ 0.01, *** *p* ≤ 0.001) or the 2-sided Dunnett’s test (‡ *p* ≤ 0.05, ‡‡‡ *p* ≤ 0.001).

To assess the abiotic reactivity, the so-called ferric reduction ability of serum (FRAS) assay was applied. This assay is based on the measurement of a mass-metric Biological Oxidative Damage (mBOD) of nanomaterials due to their oxidative potential by the reduction of human blood serum [34]. For each NP and NW, a dose-response was carried out and one concentration close to ~20% of maximum NM oxidative potential was selected for the evaluation of ion contribution (Figure 2).

After incubation of the Cu NP in the FRAS assay media for the duration of the assay at a concentration of 0.04 g/L (~20% of the measured Cu NP oxidative potential), the actual Cu ion concentration was determined, being 11 mg/L. The ion oxidative potential was more than two times and thus significantly lower (18,048 ± 2863 nmol TEU/L) than the response induced by the total Cu NP (49,783 ± 644 nmol TEU/L); therefore the reactivity of Cu NP at 0.04 g/L was predominately assigned to the particle with a steep dose-response curve. Similarly, CuO NP presented high reactivity which was dominated by the particle itself (Figure S3A). The ion contribution of Cu NW was tested at a concentration of 0.1 g/L and the actual Cu ion concentration detected was 6.6 mg/L. The reactivity of Cu NW (0.1 g/L) originated completely from ions (Figure 2A). Moreover, FRAS mBOD values were calculated at concentrations of 0.22 g/L as 842 ± 10 and 922.0 ± 5.4 nmol TEU/mg for Cu NP and NW respectively, representing very high reactivity for both forms.

The ion contribution for Ni NP and NW was examined at a concentration of 15 g/L. Ni ion concentrations were determined to be 105 mg/L and 31 mg/L, respectively. Both Ni ions and NPs contributed significantly to the reactivity of Ni NP at 15 g/L (Figure 2B), with values of 23,897 ± 1470 nmol/L TEU (ions) and 30,063 ± 441 nmol/L TEU (particles). The mBOD values at concentration 15 g/L are for Ni NP 2.00 ± 0.03 nmol TEU/mg and for NW 0.50 ± 0.01 nmol TEU/mg. Although the difference is metrologically significant, the values are very similar in comparison to the dynamic range of the assay as exemplified by the values of the Cu-based materials.

Since TiO_2_ NP and NW were insoluble materials, ion contribution to the reactivity was not considered. Similar mBOD values at a concentration of 15 g/L were calculated for both NP: 1.20 ± 0.09 and NW: 1.5 ± 0.22 nmol TEU/mg (not all replicates were useful for the NW, and the error of the worst triplicate in the concentration series is given). Again, the difference is metrologically significant, but the values are very similar in comparison to the dynamic range of the assay.

Moreover, the reactivity of CeO_2_ and Ag NP was evaluated (Figure S3B,C). As an insoluble material, CeO_2_ presented very low reactivity (mBOD: 1.90 ± 0.09 nmol TEU/mg at conc. 5.6 g/L). Ag NP showed intermediate reactivity with a mBOD value of 22.3 ± 0.2 nmol TEU/mg (at conc. 3.0 g/L).

### 3.4. Cell Viability and Bioavailability

For all cellular experiments doses are stated as µg/mL to facilitate comparison between NP and NW, since the parameter of the deposited dose was only available for NP and not for NW as described in Section 3.1.

#### 3.4.1. Cell Viability

**Figure 3:**
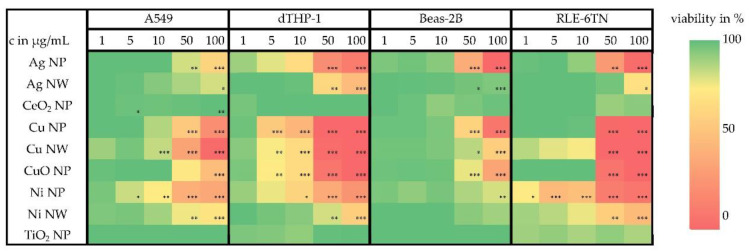
Impact of nanomaterials on the ATP content of A549, Beas-2B, RLE-6TN, and dTHP-1 cells after incubation with five different doses of the nanomaterials. The ATP content of incubated samples was normalized to an untreated control. Significantly different from negative controls: * *p* ≤ 0.05, ** *p* ≤ 0.01, *** *p* ≤ 0.001 (ANOVA-Dunnet’s *t-*test).

Cell viability was investigated in four different cell lines, namely three lung epithelial cell lines (A549, Beas-2B and RLE-6TN (rat)) and one cell line with macrophage-like properties (dTHP-1). For each nanomaterial, five different doses were chosen and their impact on the ATP content was assessed. Furthermore, RCC after incubation with three different doses was analyzed. (Figure S4). As shown in the overview provided in Figure 3, all materials which were soluble in lysosomal fluid (Cu, CuO, Ni) showed a dose-dependent cytotoxic effect in all investigated cell lines. Additionally, a dose-dependent decrease of the viability of the cells was seen after incubation with the Ag-based materials, even though an ion release in the lysosomal fluid was not observed for this material. The dTHP-1 cells, as a cell culture model with macrophage-like properties, revealed the most sensitive reaction to all lysosomal-soluble nanomaterials. Comparing NP and NW of the same material at the same concentrations, with the exception of Cu-based materials, the application of NW resulted in a less pronounced cellular toxicity. For the insoluble materials, TiO_2_ NP and CeO_2_ NP no decrease in viability was observed, even for the highest applied dose of 100 µg/mL.

#### 3.4.2. Bioavailability

**Figure 4:**
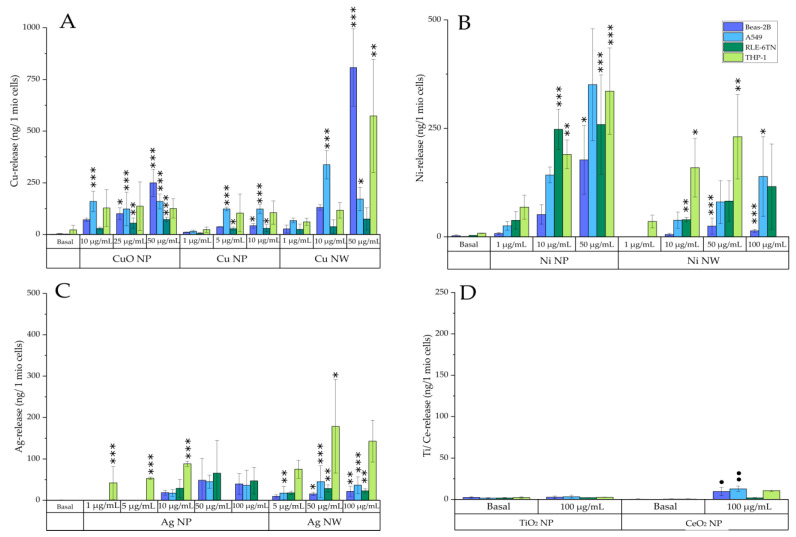
Bioavailability of Cu- (**A**), Ni- (**B**), Ag- (**C**), and Ce- and Ti-based materials (**D**) in A549 (light blue), Beas-2B (purple), RLE-6TN (dark green), and dTHP-1 (light green) cells. Bioavailability is displayed as ion release in ng/10^6^ cells. Cells were incubated with nanomaterials for 24 h in their corresponding cell culture media. Depicted are the means of three independently performed experiments ± standard deviation. Statistics were performed using either ANOVA-Dunnett’s test (* *p* ≤ 0.05, ** *p* ≤ 0.01, *** *p* ≤ 0.001) or the unpaired *t* test (• *p* ≤ 0.05, •• *p* ≤ 0.01) to compare differences from basal concentration.

Bioavailability of all nanomaterials was analyzed after 24 h incubation with three different concentrations (Figure 4) in four different cell lines (A549, Beas-2B, RLE-6TN, and dTHP-1). Doses were chosen in preliminary experiments and normalized to low, mid, and high cytotoxic effects. Cells were incubated with nanomaterials for 24 h in their respected cell culture media. Depicted are the means of three independently performed experiments ± standard deviation.

All materials which revealed a solubility in ALF (pH 4.5) also showed a dose-dependent increase of the intracellular ion release. The basal Cu content within the epithelial cell lines (A549, Beas-2B, RLE-6TN) was around 2–3 ng/10^6^ cells (15 µM). Regarding the dTHP-1 cells, a basal Cu concentration of 20 ng/10^6^ cells (30 µM) was observed. After incubation with the CuO NP, the intracellular content of released copper ions increased up to 250 ng/10^6^ cells (1700 µM), being comparable for all cell lines except the RLE-6TN cells. Here, only a small increase of intracellular copper content after incubation with the CuO NP was observed (70 ng/10^6^ cells (600 µM)). Comparing Cu NP and NW, the bioavailability of the Cu NW was considerably higher, especially in Beas-2B cells (up to 800 ng/10^6^ cells (5500 µM)). This observation could be explained by the higher dissolution rate of the Cu NW in cell culture media used for Beas-2B cultivation (shown in Figure S5) and therefore a simultaneous uptake of Cu ions in Beas-2B cells. Besides Beas-2B cells, dTHP-1 cells also exerted a strong release of Cu ions after incubation of the Cu NW, with a maximum of 570 ng/10^6^ cells (3157 µM).

For both Ni-based materials a strong dose-dependency in intracellular bioavailability was observed. Basal Ni concentrations ranged around 1–3 ng/10^6^ cells (3–10 µM) for all epithelial cells and 7 ng/10^6^ cells (26 µM) for the dTHP-1 cells. After incubation with 10 µg Ni NP/mL, intracellular Ni content increased up to 250 ng/10^6^ cells (3000 µM). In comparison, the bioavailability of Ni NW was much lower. Intracellular Ni contents up to 44 ng/10^6^ cells (485 µM) were seen for two epithelial cell lines (A549 and RLE-6TN) after an incubation dose of 10 µg Ni NW/mL After treatment with 50 µg Ni NW/mL and higher, Ni-ion content increased up to 140 ng/10^6^ cells (950 µM). Regarding the Ni content in the dTHP-1 cells at an incubation dose of 10 µg Ni NW/mL, intracellularly dissolved Ni was found to be four times higher (160 ng/10^6^ cells (600 µM)) compared to that of all epithelial cells. For Beas-2B cells only a low bioavailability was observed after incubation with Ni NW, leading to a maximum intracellular Ni content of 13 ng/10^6^ cells (90 µM). A lower bioavailability of Ni in the Beas-2B cells was also observed after incubation with the Ni NP. Solubility of the Ni-based materials in cell culture media was very low, leading to a maximum dissolution rate of 4% in all cell culture media used in this study (Figure S5). Thus, the intracellular bioavailability of these materials can be correlated to the uptake of undissolved materials. Moreover, no differences in the solubility between the different cell culture media were seen. Therefore, differences in the intracellular bioavailability of the Ni-based materials appear to be dependent on the cell lines and their specific properties.

In acellular investigations, no dissolution of the Ag-based materials in ALF was seen. However, bioavailability was observed in the cellular studies. Incubation of the Ag NP resulted in a weak dose-dependent increase of the intracellular Ag content of up to 47 ng/10^6^ cells (180 µM) at an incubation dose of 100 µg/mL for all epithelial cell lines. In contrast, Ag content in the dTHP-1 cells was much higher, reaching an intracellular Ag ion release of 94 ng/10^6^ cells (175 µM) at 10 µg Ag NP/mL Incubation of the Ag NW led to a maximum Ag ion release of 34 ng/10^6^ cells (102 µM) at the maximum dose of 100 µg/mL for the epithelial cells, whereas Ag ion content of the dTHP-1 cells was found to be around five times higher (150 ng/10^6^ cells (330 µM)) after an incubation dose of 100 µg Ag NW/mL. For the insoluble materials TiO_2_ NP and CeO_2_ NP no bioavailability was seen even after treatment with the highest dose of 100 µg/mL.

#### 3.4.3. Intracellular Distribution

**Figure 5:**
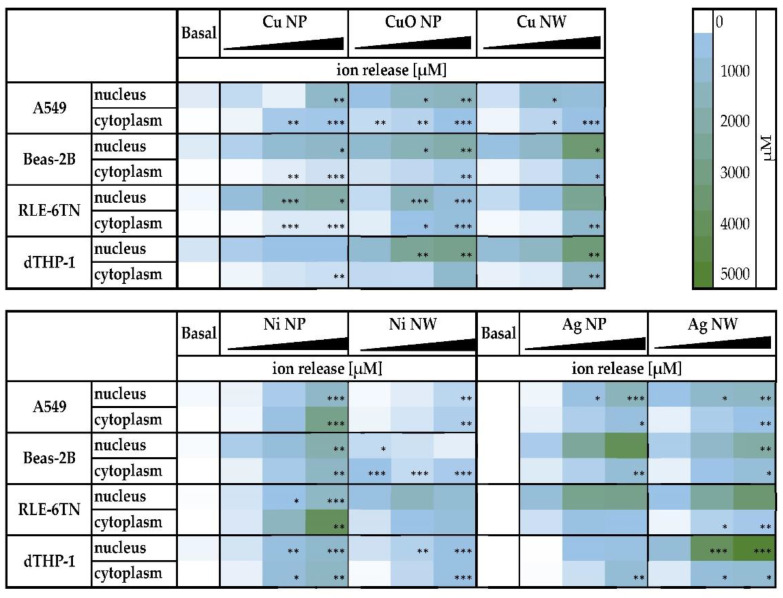
Intracellular distribution in cytoplasm and nucleus of A549, Beas-2B, RLE-6TN and dTHP-1 cells after incubation with three doses metal-based nanomaterials. Significantly different from basal concentration: * *p* ≤ 0.05, ** *p* ≤ 0.01, *** *p* ≤ 0.001 (ANOVA-Dunnet’s *t-*test).

Additionally, the intracellular distribution of all bioavailable materials was investigated by fractionating the cells into the soluble fractions of the cytoplasm and nucleus (Figure 5). Since treatment doses vary between the different materials, they are stated in Table S2. A dose-dependent increase of ion concentrations was seen for all of the materials in the cytoplasm as well as in the nucleus. For the Cu-based materials, a strong accumulation of intracellular dissolved Cu ions was found within the nucleus of all cell lines, reaching concentrations of 1 mM and higher. As already observed for the cellular bioavailability, the compartment-specific Cu concentration was much more pronounced after treatment with Cu NW when compared to the particulate Cu-based materials. Regarding the Ag-based materials, a nuclear accumulation was evident in all cell lines. Here, concentrations up to 4 mM were observed after incubation of Beas-2B cells with Ag NP and after applying Ag NW on dTHP-1 and RLE-6TN cells. For the Ni-based materials, a lower concentration of released Ni ions was found in the nucleus as compared to the cytoplasm of all cell lines.

## 4. Discussion

In this study, nine different particulate or fibrous metal-based nanomaterials were investigated with respect to their physicochemical properties and solubility behavior in acellular fluids of different pH values. Furthermore, the cytotoxicity and intracellular bioavailability of the materials in four different cell lines relevant for inhalative exposure was determined. To the best of our knowledge, this is the first study systematically comparing the impact of different particulate and fibrous metal-based materials on all of these parameters in parallel, exerting some quantitative differences between nanomaterial shapes, but a more distinct impact of the respective metal species under investigation.

Concerning the acellular investigations, for all materials, solubility in AAF (pH 7.4) was not apparent or considered to be low. This suggests that the analyzed materials do not dissolve in the extracellular matrix of the respiratory tract which is in accordance with previous studies [24]. Therefore, a nanomaterial-cell interaction within the lung can be postulated. However, since nanomaterials are taken up via endocytosis and are subsequently transported to lysosomes, dissolution in this acidic environment appears to be relevant. This may result in higher solubility in this acidic cellular compartment, with potential intracellular metal ion release and thus potential metal-ion derived cellular toxicity. Therefore, two different approaches were chosen to determine solubility under acidic conditions and compared, namely, the static dissolution in artificial lysosomal fluid (ALF) and a dynamic dissolution approach in phagolysosomal simulant fluid (PSF), both pH 4.5. The dynamic approach was chosen additionally, since the lung is not a static system and dissolved ions are transported quickly to other compartments, rendering it as a more realistic approach [36,37]. Furthermore, the dissolution by the dynamic approach is not limited by saturation conditions and therefore an underestimated dissolution rate can be prevented [38]. Regarding the different materials under investigation, even after seven days no solubility in either experimental system was observed in case of TiO_2_ NP or NW, CeO_2_ NP nor in case of Ag NP. However, some solubility was observed for Ag NW in the dynamic system as opposed to no detectable dissolution under static conditions. Far higher solubility of around 50% and above was evident in case of Ni- and Cu-based materials. Here, both Ni NP and Ni NW, as well as CuO NP, exerted higher dissolution fractions in the dynamic system, while the opposite was observed in case of Cu NP and Cu NW. Nevertheless, in all cases except for the insoluble TiO_2_, CeO_2_, and Ag NP, solubility was highly accelerated under acidic conditions, evident by both experimental approaches. These differences in nanomaterial dissolution were also reflected in structural transformation as determined by TEM in the dynamic approach.

Additionally, the oxidative potentials of NP and NW and their free ions were evaluated by utilizing the FRAS assay, which measures biological oxidant damage in serum [15]. Here, NP and NW based on the same metals (Cu NP/NW, Ni NP/NW, TiO_2_ NP/NW) demonstrated similar reactivity. However, the difference between the metals was more significant. While all Cu-based materials (NP/NW and CuO NP) exerted very high reactivity at low concentrations around and above 0.1 g/L, about 100-fold higher concentrations were required in case of TiO_2_ NP/NW, Ni NP/NW, and CeO_2_ NP to exert some but still low reactivity. With regard to the respective NP, the results confirm those obtained previously [20,39]. No such studies have been conducted for the NW analyzed within this study.

Since the respiratory tract is a complex system consisting of different cell types, the cytotoxicity and bioavailability of the nanomaterials in four different cell lines were investigated, all being relevant for the respiratory tract. Thus, three epithelial cell lines of human (A549, Beas-2B) or rat (RLE-6TN) origin, as well as a cell line with macrophage-like properties (differentiated THP-1) were applied. To assess the cytotoxicity, two parameters were chosen, namely RCC and ATP content, which were determined in all four cell lines. Based on the outcome, bioavailability studies were conducted at low, mid, and high cytotoxic doses of the respective materials, as stated in Table S2. To distinguish intracellular bioavailability from particles potentially stuck to the outer cell membrane, and to further discriminate between cytoplasmic and nuclear metal ion concentrations, two different fractionation protocols were applied as published previously [15,17]. Briefly, to assess bioavailability in whole cells, the cell membrane with potential material residues was separated by cell lysis followed by a centrifugation step. Besides the bioavailability in the whole cell, metal-ion concentrations in the cytoplasm and the nucleus were investigated. Here, cells were separated into the soluble fractions of cytoplasm and nucleus. In both approaches, metal-ion concentration was determined by atomic absorption spectrometry or ICP-MS afterwards.

In general, with the notable exception of Ag-based materials, both cytotoxicity as well as bioavailability reflected the acellular dissolution rates in physiological lysosomal media (pH 4.5), since materials that exhibited an acellular dissolution also showed a dose-dependent cytotoxicity and bioavailability within all tested cell lines. Here, highly elevated concentrations were seen in the cytoplasm and the nucleus; particularly high concentrations in the nucleus were found in the case of Cu- and Ag-based materials, reaching millimolar concentrations.

TiO_2_ and CeO_2_ NP, which were insoluble in acellular lysosomal fluid, also showed neither cytotoxicity nor intracellular metal ion release. This is in agreement with a previous study showing that resorbed TiO_2_ NP remained within the phagosomes of the cells without measurable ion release in the cytoplasm and caused no cellular toxicity [40].

A good correlation between solubility in artificial lysosomal fluids, cytotoxicity, and intracellular bioavailability was also evident for Cu- and Ni-based materials, showing some cell line depending differences. For CuO NP, a pronounced and dose-dependent bioavailability was seen in the Beas-2B cells, followed by A549 and dTHP-1 cells. The correlation between the solubility at a low pH and intracellular bioavailability was already described for CuO NP in two previous studies, where the dissolution in ALF with the intracellular bioavailability in A549 and Beas-2B cells was compared [15,17]. Interestingly, the bioavailability of Cu NP in the Beas-2B cells, when compared to the A549 cells, was much lower. Comparing Cu NP and Cu NW at the same doses, a higher bioavailability was seen for Cu NW, however, the toxic effects of Cu NP and Cu NW were comparable. Additionally, high concentrations of released ions from Cu NW were found in the nuclei. The observation of higher Cu ion concentration within nuclei by Cu NW compared to Cu NP may result in a higher genotoxic potential of the fiber-shaped material due to the redox potential of Cu ions. However, this hypothesis needs to be further evaluated in subsequent studies.

After incubation with Ni NP, a dose-dependent intracellular nickel ion release was evident in all cell lines, with somehow less pronounced uptake in Beas-2B cells. This is in agreement with results presented previously by Capasso and colleagues, who demonstrated that the uptake of NiO NP in A549 cells is mainly endocytosis-related, while there was no evidence for endocytotic uptake of NiO NP in Beas-2B cells [41]. The same tendency was seen in the case of Ni NW, with lower levels of deliberated metal ions in all cell lines, possibly due to the branched structure of the fibers. Recent studies have already shown that Ni NW are taken up by different cell types, such as fibroblasts [42], colon cancer cells [43], and macrophages [44] causing different toxic effects. However, this study offers a quantitative comparison of the bioavailability of Ni NP and NW in different cell lines, which has, to that extent, not been published. Interestingly, Ni NW exhibited a higher bioavailability in dTHP-1 cells when compared to the epithelial cell lines. This indicates that macrophages rapidly start to take up nanowire via phagocytosis, which has already been observed in vivo [45]. Despite the fact that high concentrations of Ni ions were also found in the nucleus of all cells after incubation with the Ni materials, it can be stated that the intracellular released Ni ions mainly remain in the cytoplasm of all cell lines. This observation strengthens results reported by Schwerdtle and colleagues, who investigated the impact of NiO MP in A549 cells [46].

One very interesting example of differences in bioavailability observed in cells and suggested solubility from acellular studies is the case of Ag-based materials. While neither Ag NP nor Ag NW showed considerable ion release, even in acidic artificial lysosomal media, Ag NP, as well as Ag NW, revealed an intracellular bioavailability at all applied concentrations. The observed intracellular bioavailability is in agreement with recent studies [24,47]. Intracellular metal ion release was highest in dTHP-1 cells, with even higher metal ion concentrations in the nucleus when compared to the cytoplasm. The discrepancy between the acellular solubility and intracellular bioavailability appears to be unique for Ag-based materials and may be explained by the fact that silver forms insoluble secondary structures due to its affinity to S- and Cl-groups [48]. These secondary structures are not quantifiable by the static solubility approach used in this study, and may not be fully quantifiable with the dynamic approach either, even though some solubility was observed in the latter test system. Thus, it cannot be excluded that also in the acellular studies, there was a release of Ag ions which bound rapidly to buffer components resulting in the formation and precipitation of these insoluble secondary particles. Within the cell, however, Ag ions may be released, leading to a dynamic equilibrium between cellular reactants, and being quantifiable within the soluble fractions of the respective compartments.

## 5. Conclusions

While only minor differences were seen for acellular dissolution and abiotic oxidative reactivity detected by the FRAS assay when comparing NP and NW of the same metal, their reactivity and dissolution are mostly driven by the respective metal under investigation. High solubility in acidic fluids, as models for the lysosomal environment, and pronounced reactivity was seen for Cu-based particulate and fibrous materials. Similarly, high solubility but moderate reactivity was seen for Ni NP and NW. Interestingly, in the case of Ag, no dissolution in acellular fluids was observed, probably due to the formation of insoluble secondary structures; however, an intermediate oxidative reactivity was seen for the Ag NP. CeO_2_- and TiO_2_-based materials exhibited no acellular dissolution and no oxidative reactivity. The dissolution behavior of the metal-based nanomaterials was strongly reflected in cellular toxicity and intracellular bioavailability. Thus, CeO_2_- and TiO_2_-based materials showed neither cytotoxicity nor intracellular bioavailability in either cell line, while the bioavailability which was seen for the soluble materials also correlated with the cytotoxicity of these materials. Cytotoxic effects appear to be due to intracellular dissolved metal ions followed by a metal ion overload, and not due to nanomaterial-cell interactions. This is in line with the proposed Trojan-horse type mechanism [13,49]. An interesting exception was seen in the case of Ag-based materials; here the acellular dissolution was not predictive for its cellular toxicity and bioavailability. This may be due to the formation of secondary particles formed after the dissolution of the nanomaterials, which likely precipitate in acellular systems and thus remain undetectable in the soluble fraction, but which may add to the soluble and thus bioavailable fraction in the cellular system. Concerning the different cell lines applied, differences in toxicity and bioavailability were metal-dependent, with no common pattern across the metals. In the case of Ni NW and Ag NW, a comparatively high bioavailability was seen in THP-1 cells with macrophage-like properties, supporting their higher proficiency for phagocytotic uptake.

## Figures and Tables

**Table 1:** Sources of all nanomaterials included in this study.

Material	Form	Source	Name/Item Number
Ag	NP	RAS AG	Agpure W10 (NM300K)
	NW	RAS AG	ECOS HC
CeO_2_	NP	JRC	NM212
Cu	NP	Io-li-tec	NM-0016-HP
	NW	PlasmaChem	PL-CuW50
CuO	NP	BASF	CUO_1_NP_PROD *
Ni	NP	Sigma-Aldrich	577995
	NW	PlasmaChem	PL-NiW200
TiO_2_	NP	JRC	NM105
	NW	PlasmaChem	PL-TiOW50

* from Sustainable Nanotechnologies Project [26]. NP: nanoparticle; NW: nanowire.

**Table 2:** Summarized physicochemical properties of the investigated nanoparticles. d_p_: primary particle diameter determined by transmission electron microscopy, d_h_: hydrodynamic diameter, PDI: polydispersity index, SSA: specific surface area.

	Cu NP	CuO NP	Ni NP	TiO_2_ NP (NM105)	CeO_2_ NP (NM212)	Ag NP (NM300K)
**d_p_ (nm)**	55.2 ± 1.5	17.1 ± 0.4	21.4 ± 0.1	23.7 ± 0.5	21.5 ± 0.3	15.5 ± 0.04
**d_h_ (nm)**	308.2 ± 40.3	160.3 ± 42.1	388.0 ± 33.2	165.8 ± 14.2	187.0 ± 7.3	72.4 ± 10.0
**PDI**	0.23 ± 0.07	0.48 ± 0.05	0.67 ± 0.02	0.14 ± 0.01	0.20 ± 0.02	0.31 ± 0.06
**ζ-potential (mV)**	−15.3 ± 0.02	−14.8 ± 0.2	−15.7 ± 0.2	−14.8 ± 0.2	−15 ± 0.6	−11.2 ± 2.1
**SSA (m^2^/g)**	10.7 ± 0.6	47 ^#^	6.4 ± 0.3	46.2 *	27 *	N/A **
**effective density (g/cm^3^)**	1.78 ± 0.02	1.98 ± 0.03	2.54 ± 9.14	1.38 ± 0.06	1.97 ± 0.14	2.07 ± 0.14
**fraction of deposited dose in 24 h (%)**	64	56	90	22	53	27
**purity (% wt)**	98.6 ± 0.4	98.7 ± 0.81	98.7 ± 0.86	91.5 ± 061	98.5	99.3 ± 0.08

* data taken from respective JRC report [35]. ** not quantified as material is only available as dispersion. # data taken from project data (SUN-project) [26].

**Table 3:** Summarized physicochemical properties of the investigated nanowire. Length and width have been determined by scanning electron microscopy, SSA: specific surface area.

	Length (µm)	Width (nm)	ζ-Potential (mV)	SSA (nm^2^/g)	Purity (% wt)
**Cu NW**	6.3 ± 0.4	300 ± 6	−14.1	1.49	>99.5 ^#^
**Ni NW**	9.97 ± 0.29	280 ± 6	−14.5	1.61	99.1
**Ag NW**	10.6 ± 0.28	110 ± 1.6	−4.1 ± 0.1	3.47	99.1 ± 0.65
**TiO_2_ NW**	7.3 ± 0.2	0.87 ± 0.03	n.a.	n.a.	n.a.

# data from supplier. n.a.: not available; experiments were not performed due to batch-to-batch variation of TiO_2_ NW resulting in suspensions of poor quality.

**Table 4:** Summary of the solubility in biologically relevant model fluids after seven days. AAF: Artificial alveolar fluid (pH 7.4); ALF: Artificial lysosomal fluid (pH 4.5); PSF: Phagolysosomal simulant fluid (pH 4.5).

Material and Form		Static Dissolution (% Dissolved)		Dynamic Dissolution (% Dissolved)
		AAF	ALF	PSF
**Ag**	NP (NM 300K)	0.5 ± 0.1	0.2 ± 0.0	1.6
	NW	0.5 ± 0.2	0.2 ± 0.0	11
**CeO_2_**	NP (NM 212)	0.001	0.021	0.3
**Cu**	NP	12.0 ± 4.7	63.8 ± 3.4	45.5
	NW	6.0 ± 2.7	57 ± 3.1	35
**CuO**	NP	3.9 ± 1.8	57.2 ± 5.1	97.3
**Ni**	NP	3.7 ± 0.4	56.1 ± 15.5	63.2
	NW	0.9 ± 0.6	35.5 ± 10.1	94.4
**TiO_2_**	NP (NM 105)	0.002	0.022	0.3
	NW	0.001	0.021	0

## Data Availability

The data presented in this study are available on request from the first (J.W., D.A.S.) and corresponding author (A.H.) for researchers of academic institutes who meet the criteria for access to the confidential data.

## Supplementary Materials

The following supporting information can be downloaded at: https://www.mdpi.com/article/10.3390/nano12010147/s1. Table S1: Parameters used in the DG-model simulations; Table S2: Dose selection for in vitro studies; Table S3: Comparison of freshly prepared and thawed particle dispersions with regard to Z-Average and deposited dose fraction calculated using the DG model; Figure S1: Size distribution of freshly prepared and thawed particle dispersions at concentrations of 100 µg/mL in supplemented RPMI-1640; Figure S2: Impact of freshly prepared and thawed particle dispersions at concentrations of 100 µg/mL in supplemented RPMI-1640 on the deposited dose fraction applying the DG model; Figure S3: FRAS-Testing; Figure S4: Impact of nanomaterials on the relative cell count of A549, Beas-2B, RLE-6TN and dTHP-1 cells after incubation with three different doses; Figure S5: Dissolution in cell culture media.

## Abbreviations

AAF	Artificial Alveolar Fluid
ALF	Artificial Lysosomal Fluid
BSA	Bovine serum albumin
DLS	Dynamic light scattering
dTHP-1	Differentiated monocytic THP-1 cells to macrophage-like cells
FRAS	Ferric Reduction Ability of Serum
GF-AAS	Graphite Furnace Atomic Absorption Spectrometry
ICP-MS	Inductively Coupled Plasma-Mass Spectrometry
NP	Nanoparticle
NW	Nanowire
PDI	Polydispersity index
PSF	Phagolysosomal Simulant Fluid
RCC	Relative Cell Count
ROS	Reactive oxygen species
REM	Raster electron microscopy
TEM	Transmission electron microscopy
