# Supplementary material for: Comparison of Metal-Based Nanoparticles and Nanowires: Solubility, Reactivity, Bioavailability and Cellular Toxicity
Source: Nanomaterials (Basel). 2021 Dec 31;12(1):147. doi: 10.3390/nano12010147 (PMC8746854; doi:10.3390/nano12010147)
Supplement: Supplementary file 1 [file nanomaterials-12-00147-s001.zip › nanomaterials-1497961-supplementary.pdf]

nanomaterials

Supplementary files

Comparison of Metal-Based Nanoparticles and Nanowire: Solubility, Reactivity, Bioavailability and Cellular Toxicity

Johanna Wall [1,†], Didem Ag Seleci [2,†], Feranika Schworm [1], Ronja Neuberger [1], Martin Link [1], Matthias Hufnagel [1], Paul Schumacher [1], Florian Schulz [3], Uwe Heinrich [4], Wendel Wohlleben [2] and Andrea Hartwig [1,*]

[1] Department of Food Chemistry and Toxicology, Faculty of Chemistry and Biosciences, Institute of Applied Biosciences, Karlsruhe Institute of Technology (KIT), 76131 Karlsruhe, Germany; Johanna.Wall@kit.edu (J.W.); fschworm@gmail.com (F.S.); Ronja.Neuberger@kit.edu (R.N.); Martin.Link@kit.edu (M.L.); Matthias.Hufnagel@gmail.com (M.H.); Paul.Schumacher@kit.edu (P.S.)
[2] BASF SE, 67063 Ludwigshafen, Germany; Didem.Ag-Seleci@basf.com (D.A.S.); wendel.wohlleben@basf.com (W.W.)
[3] Fraunhofer ITEM, 30625 Hannover, Germany; Florian.Schulz@item.fraunhofer.de
[4] ToxConsultant, 30625 Hannover, Germany; uwe.heinrich1512@gmail.com
* Correspondence: Andrea.Hartwig@kit.edu
† These authors contributed equally to this work.

Supplementary Table S1. Parameters used in the DG-Model simulations.

Parameter	Used value
Solvent dynamic viscosity	0.00081 Pa*s
Media density (supplemented RPMI-1640)	1.008368 g/cm³
Temperature	37 °C
Effective density	Mean of three replicates, values described in Table 1 [g/cm³]
Size distribution	Measured values via DLS after 1 h incubation at 37 °C, mean of three replicates as shown in Figure S1 [nm]
Media height	3 mm (for 96 well plate)
Initial material concentration	0.1 mg/cm³
Simulation time	24 h
Gravity	1 g
Height of simulation sub-compartment	0.005 mm
Simulation time interval	0.5 s
**Output Parameters**	
Output data time intervals	30 min
Output compartment height	0.005 mm
Adsorption dissociation constant (K_d)	$1x10^{-9}$ (Adhesive Boundary conditions)

Supplementary Table S2. Dose selection for *in vitro* studies. *adapted dose for dTHP-1 cells

Material	Dose (µg/mL)		
	Low	Mid	High
**CeO_2 NP**	1	50	100
**TiO_2 NP**	1	50	100
**CuO NP**	10	25	50
**Cu NP**	1	5	10
**Cu NW**	1	10	50
**Ni NP**	1	10	50
**Ni NW**	10/1*	50/10*	100/50*
**Ag NP**	10/1*	50/5*	100/10*
**Ag NW**	5	50	100

Supplementary Table S3. Comparison of freshly prepared and thawed particle dispersions with regard to Z-Average and deposited dose fraction calculated using the DG model

	Z-Average (nm)		Deposited dose fraction (%)	
	Fresh	Thawed	Fresh	Thawed
**Cu NP**	308.2 ± 40.3	299.4 ± 53.3	64	61
**CuO NP**	160.3 ± 42.1	157.4 ± 19.6	56	54
**Ni NP**	393.8 ± 29.5	537.0 ± 52.9	92	86
**NM105**	165.8 ± 14.2	159.9 ± 3.1	22	21
**NM212**	187.0 ± 7.3	192.7 ± 2.0	53	53
**NM300K**	72.4 ± 10.0	59.2 ± 18.2	27	23

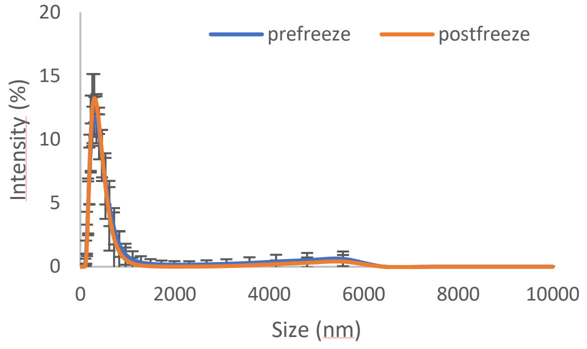

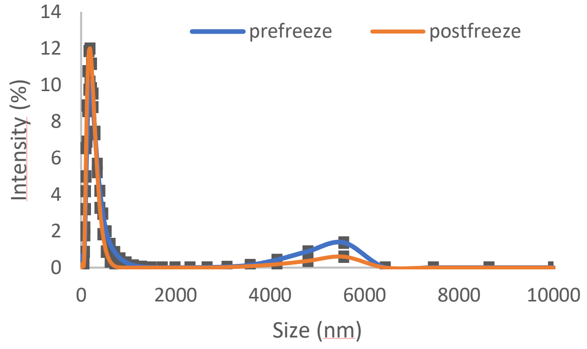

(A) (B)

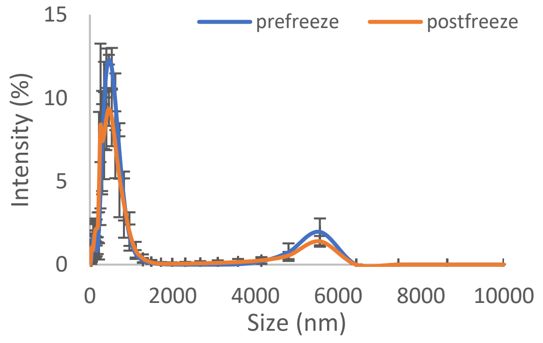

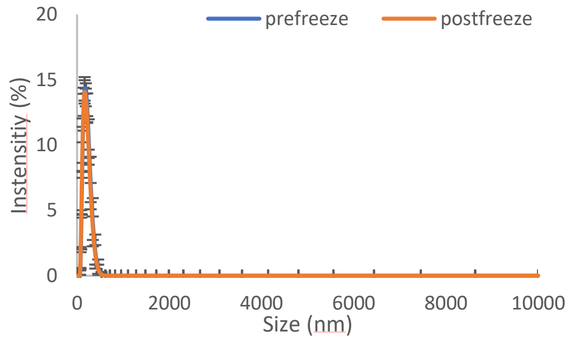

(C) (D)

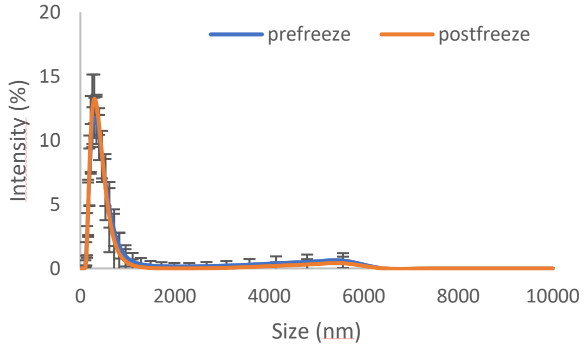

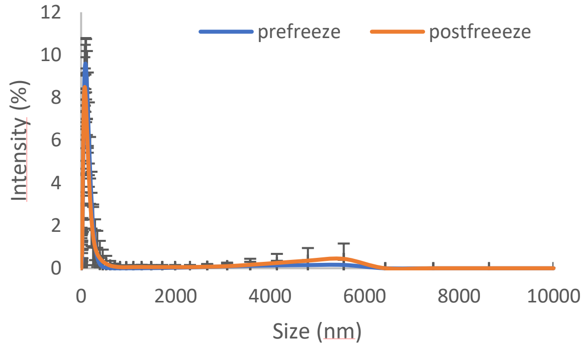

Supplementary Figure S1. Size distribution of freshly prepared (prefreeze, blue) and thawed (postfreeze,red) particle dispersions at concentrations of 100 µg/mL in supplemented RPMI-1640. A: Cu NP, B: CuO NP, C: Ni NP, D: NM105, E: NM212, F: NM300K.

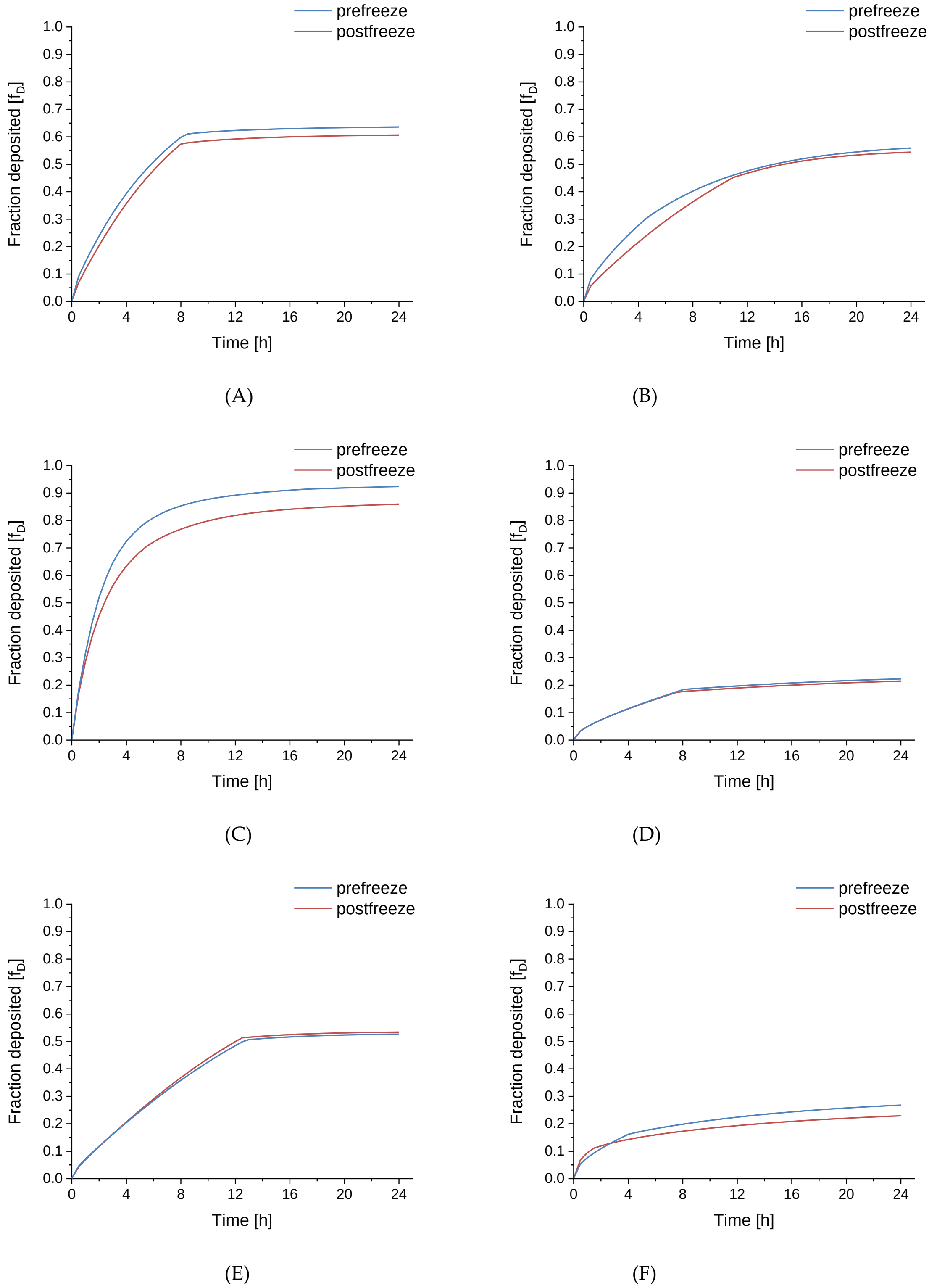

Supplementary Figure S2. Impact of freshly prepared (prefreeze, blue) and thawed (postfreeze, red) particle dispersions at concentrations of 100 µg/mL in supplemented RPMI-1640 on the deposited dose fraction applying the DG model. A: Cu NP, B: CuO NP, C: Ni NP, D: NM105, E: NM212, F: NM300K.

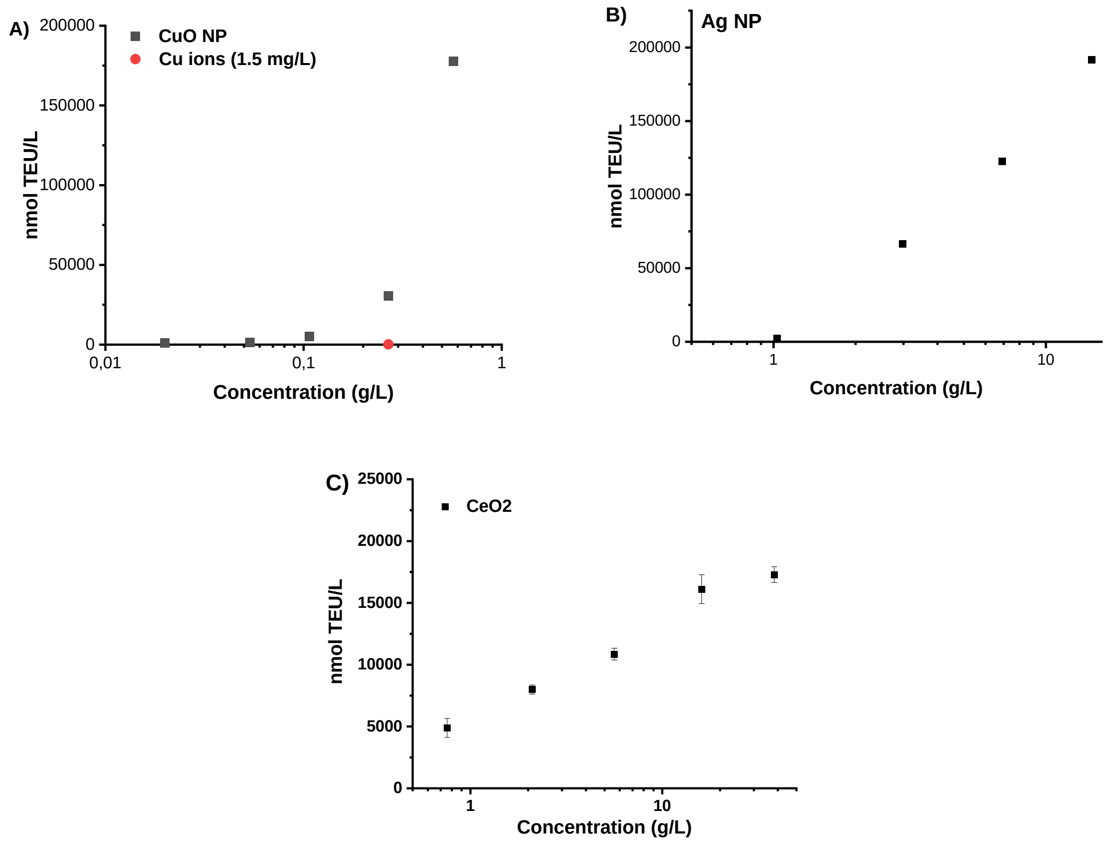

Supplementary Figure S3. FRAS testing A) CuO NP, CuO NP_ions B) Ag NP C) CeO_2 NP.

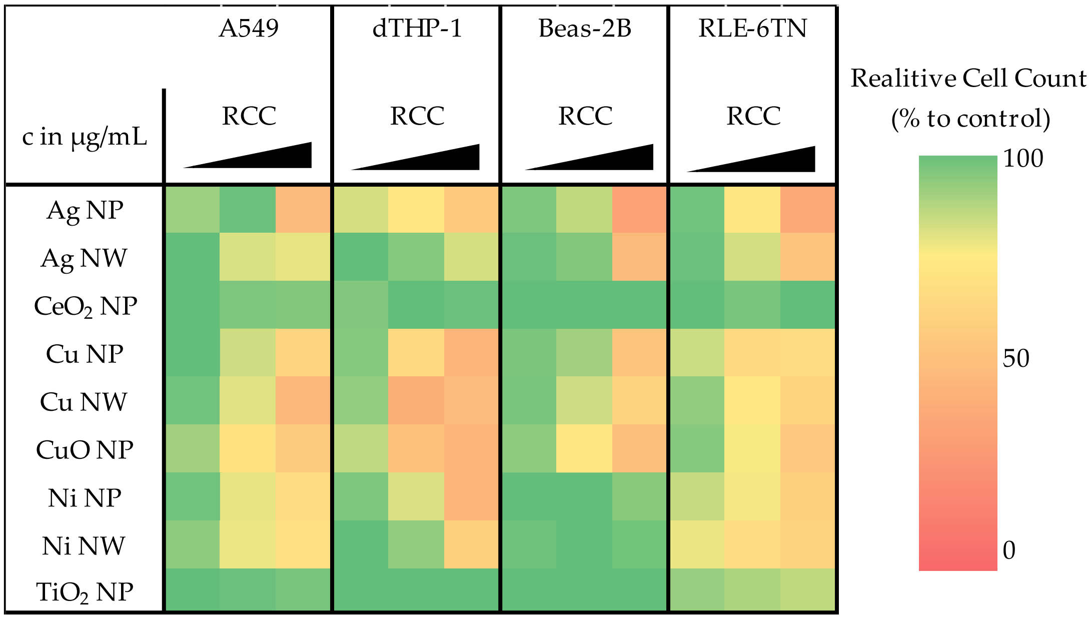

Supplementary Figure S4. Impact of nanomaterials on the relative cell count (RCC) of A549, Beas-2B, RLE-6TN and dTHP-1cells after incubation with three different doses. Doses were chosen in preliminary experiments and normalized to low, mid and high cytotoxic effects. Since doses vary between the different materials, they are stated summarized in Tab. S3.

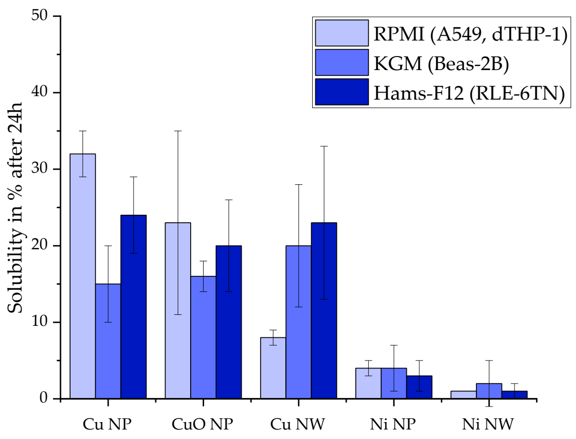

Supplementary Figure S5. Dissolution in cell culture media. The solubility was measured after 24 h in the cell culture media used for *in vitro* studies.
